# The Importance of Isomorphism for Conclusions about Homology: A Bayesian Multilevel Structural Equation Modeling Approach with Ordinal Indicators

**DOI:** 10.3389/fpsyg.2016.00289

**Published:** 2016-03-02

**Authors:** Nigel Guenole

**Affiliations:** ^1^Goldsmiths, University of LondonLondon, UK; ^2^IBM Smarter WorkforceLondon, UK

**Keywords:** isomorphism, homology, multilevel structural equation modeling, ordinal indicators

## Abstract

We describe a Monte Carlo study examining the impact of assuming item isomorphism (i.e., equivalent construct meaning across levels of analysis) on conclusions about homology (i.e., equivalent structural relations across levels of analysis) under varying degrees of non-isomorphism in the context of ordinal indicator multilevel structural equation models (MSEMs). We focus on the condition where one or more loadings are higher on the between level than on the within level to show that while much past research on homology has ignored the issue of psychometric isomorphism, psychometric isomorphism is in fact critical to valid conclusions about homology. More specifically, when a measurement model with non-isomorphic items occupies an exogenous position in a multilevel structural model and the non-isomorphism of these items is not modeled, the within level exogenous latent variance is under-estimated leading to over-estimation of the within level structural coefficient, while the between level exogenous latent variance is overestimated leading to underestimation of the between structural coefficient. When a measurement model with non-isomorphic items occupies an endogenous position in a multilevel structural model and the non-isomorphism of these items is not modeled, the endogenous within level latent variance is under-estimated leading to under-estimation of the within level structural coefficient while the endogenous between level latent variance is over-estimated leading to over-estimation of the between level structural coefficient. The innovative aspect of this article is demonstrating that even minor violations of psychometric isomorphism render claims of homology untenable. We also show that posterior predictive *p*-values for ordinal indicator Bayesian MSEMs are insensitive to violations of isomorphism even when they lead to severely biased within and between level structural parameters. We highlight conditions where poor estimation of even correctly specified models rules out empirical examination of isomorphism and homology without taking precautions, for instance, larger Level-2 sample sizes, or using informative priors.

## Introduction

Researchers in the social sciences deal with phenomena that are inherently multilevel. In management research, for instance, individual employees are embedded in teams, teams comprise business units, and business-units form organizations. In educational psychology, students are nested within classrooms, classrooms are nested within schools, and schools are nested in school districts. In these research settings it is commonly the case that intrinsically micro level attributes of individuals are measured and that these measurements are aggregated for analysis to the *meso* (e.g., classrooms or teams) or *macro* levels (e.g., schools or firms). The newly formed higher-level constructs can be related to other variables that are similarly aggregated or to variables that were measured directly at the higher level of aggregation. Such analyses are considered multilevel in nature. This article focuses on structural relations between constructs measured at some lower level of analysis, (i.e., a micro level, such as the individual) and aggregated to some higher (i.e., meso or macro) level. We use the terms meso and macro to represent any level of aggregation of interest that is higher than the level at which the construct was measured.

Parsimony and generalizability are important goals in statistical modeling (Forster, 2000). With this perspective in mind, a natural question to ask in multilevel contexts is whether the constructs measured at lower micro levels have similar conceptual interpretations to their aggregated counterpart constructs. It is also natural to inquire about whether nomological (i.e., structural) relationships between psychological attributes at the micro level are equivalent to nomological relationships observed at the aggregated level. Should constructs have similar measurement interpretations and similar nomological relations with other variables at micro and meso or macro levels of a multilevel model, the multi-level model can be considered more parsimonious than one that specifies different construct interpretations and structural relations across levels. It can also be considered a model that generalizes across levels of analysis. Equivalence of construct meaning in a psychometric sense for psychological constructs across micro, meso and macro levels is referred to as *isoporphism* in the psychometric literature (Muthén, 1994; Dyer et al., 2005; Tay et al., 2014), while equivalence of nomological relations across levels is referred to as *homology* (Chan, 1998; Morgeson and Hofmann, 1999; Chen et al., 2005). Tay et al. (2014) discuss three further important advantages bestowed on multilevel research designs incorporating isomorphic measurement models (i.e., measurement equivalence across levels of analysis). First, individuals within the higher-level units represent a tangible instantiation of the higher-level concept, and vice versa. Second, concern about anthropomorphizing individual level attributes at the team level or inappropriate generalizing team level concepts to individuals is removed. Finally, these authors suggest isomorphism permits generalizing theories developed at one level of analysis for explanation at another level of analysis. Overall, isomorphism, or cross level invariance in multilevel modeling, is an important topic in educational and organizational sciences.

### Similarities in approaches to multi-group equivalence and multi-level equivalence

Early thinking about isomorphism and homology in the multilevel literature bears resemblance to early thinking about the relationship between measurement equivalence and relational equivalence in single level contexts. For some time, researchers studied whether structural relationships between variables were equivalent across groups without first examining measurement equivalence. Today, however, it is recognized that measurement invariance is an important pre-requisite for interpreting results of analyses of structural invariance (Drasgow, 1982, 1984; Millsap, 1995, 1998; Chen, 2008). Several articles in a special issue on measurement invariance in this journal edited by van de Schoot et al. (2015b) illustrate the necessity of and steps for correcting for non-invariant measurement indicators when structural relations across groups are the focus on research interest (e.g., Guenole and Brown, 2014; Hox et al., 2015).

Similarly, earlier work on homology suggested that the structural equivalence across levels could be investigated based on what might be referred to as loose evidence of construct isomorphism. For instance, Chen et al. (2005, p. 375) stated “We do, however, take the position that the coupling of construct meaning across levels is first and foremost a theoretical issue and that measures of the construct at different levels need not be psychometrically equivalent (i.e., they need not be isomorphic).” Chen et al.'s (2005) rationale was that true isomorphism is not possible for psychological constructs because the processes that led to the emergence of constructs at each level differ. These processes tend to be a blend of biological and psychological at the level of the individual, but primarily sociological at higher levels of aggregation. However, there is a growing realization today that informal approaches to isomorphism are better replaced by formal modeling approaches that test this assumption (e.g., Muthén, 1994; Chan, 1998; Boomsma et al., 2013; Kozlowski and Klein, 2000; Bliese et al., 2007; Zyphur et al., 2008) and that the process of emergence should be considered separately from issues psychometric isomorphism (Tay et al., 2014). Indeed, this position is similar to that taken by applied measurement practitioners who wish to eliminate items that show measurement bias without too much regard for the processes that led to the non-invariance.

Recent research on isomorphism, using the multilevel structural equation modeling (MSEM) technique, has focused on methods to examine equivalence across clusters (i.e., cluster bias) that sit within levels at both lower and higher levels of aggregation as well as the relationship between measurement invariance across groups within levels and invariance across levels (Jak et al., 2013, 2014a; Ryu, 2014, 2015; Kim et al., 2015). In addition, MSEM research has witnessed a considerable and necessary focus on research design requirements for accurate estimation of measurement and structural parameters in MSEM under different estimation methods (e.g., Hox et al., 2012, 2014). However, there has been little or no research into the consequences of what we argue below is a potentially convenient misspecification in MSEMs, i.e., small to moderate violations of invariance across levels (i.e., isomorphism) for relations with external variables (i.e., homology). This is a notable gap in the context of MSEM, which is widely agreed as one of the most rigorous methods for testing isomorphism and homology.

### A taxonomy of levels of isomorphism

Tay et al. (2014) proposed a new taxonomy of levels for multilevel isomorphism. These authors differentiate the following levels of configural and metric isomorphism, or “across level” measurement invariance. Strong configural isomorphism exists when the same number of factors exists on the within and between levels and the factor structure contains the same pattern of fixed and free loadings. When the same number of factors exists on multiple levels of analysis but the pattern of fixed and free factor loadings is not the same, weak configural invariance is said to exist. It is possible, and in fact common, for fewer factors to be required at higher levels of analysis and for the higher-level model to exhibit an entirely different pattern of loadings. In this case, there is no basis for claims of isomorphism. However, if some of the factors are reproduced with the same zero non-zero loadings patterns, partial configural isomorphism is said to exist. Strong metric isomorphism is said to exist when a model that shows strong configural isomorphism also exhibits equivalent loadings across levels of analysis. Weak metric isomorphism exists when the rank ordering of the loadings of items is equivalent across levels but the precise magnitudes are not. If even the rank ordering of loadings is not equivalent across levels, there is no basis for claiming metric isomorphism.

### Implications of isomorphism for homology

As yet, no consideration has been to the consequences of these levels of invariance for relations with external variables, i.e., structural relations between measurement models across levels. Investigating this issue is the goal of the current study, which can be considered an example of examining the practical consequences of convenient model misspecifications. Studies of such misspecification abound in the psychometric literature. Instances include exact vs. approximate fit in structural equation models (Hu and Bentler, 1999), whether data are “unidimensional enough” that item parameters can be considered dependable (Drasgow and Lissak, 1983; Bonifay et al., 2015) and the extent to which measurement invariance can be ignored in multiple group confirmatory factor analyses without detrimentally impacting substantive conclusions about regression between latent constructs across groups (Chen, 2008; Guenole and Brown, 2014). Similar studies have also examined the impact of model misspecification in bi-factor contexts. For example, Reise et al. (2013) examined the effect of ignoring bi-factor structures on structural parameter bias as a function of the percentage of “contaminated correlations” in the covariance matrix.

More recently, general methods have been proposed that examine the consequences of model constraints for particular parameters in models (Kuha and Moustaki, 2015; Oberski, 2014; Oberski et al., 2015) although these approaches are so far untested in the context of isomorphism in multilevel modeling. In this article, we show that absent strong evidence of metric isomorphism, evidence about structural relations across levels is rendered difficult to interpret at best and at worst uninterpretable due to bias in the estimation of structural relations. Isomorphism must be addressed before drawing conclusions about homology.

### Theoretically derived research question

The goal of the current article is to examine the implications of psychometric isomorphism for conclusions about homology in the context of MSEMs with categorical indicators. We investigate empirically whether there is any good reason to expect whether or not psychometric isomorphism (or its absence) is accurately modeled has important implications for conclusions about construct homology (i.e., the equivalence of structural relations across levels of analysis). We use a Monte Carlo experimental design to investigate what degree of psychometric *non*-isomorphism can be countenanced while still reaching accurate conclusions about psychometric evidence for homology. This is an important issue representing a trade off applied researchers primarily interested in homology will often face. If evidence of non-isomorphism is minor, the temptation could be to ignore the non-isomorphism and impose the same measurement models across levels of analysis. This would permit the claim of a consistent meaning of constructs across levels.

For instance, along with all the ensuing benefits we have discussed, this would allow researchers to say that the same psychological constructs exist across levels with the same nomological relationships instead of needing to say that similar constructs exist across levels with similar relationships with external variables. However, applied researchers would be less likely to take this course of action if imposing equivalence across levels led to inaccurate conclusions about homology, which is often a researcher's primary interest. Here we concern ourselves with the situation where researchers recognize that separate models should be estimated for each level rather than the case where researchers erroneously estimate models at one level when data are in fact multilevel. For more on the problems with this approach see Zyphur et al. (2008).

### Multilevel structural equation modeling

In this article we adopt the MSEM framework to examine our hypotheses regarding multilevel isomorphism. MSEM has several advantages that place it among the primary choices for measuring multilevel constructs (Bliese et al., 2007). For instance, MSEM allows simultaneous estimation of measurement models on within (disaggregated) and between (aggregated) levels while in parallel permitting the estimation of structural relationships between measurement models on within and between levels. In addition, a formal statistical test of model fit in the form of the likelihood ratio test is available. Widely known close fit indices provided by common software programs are available for MSEM models under frequentist and Bayesian estimation approaches. Readers may refer to Hox (2010) and Ryu and West (2009) for discussions of the adjustments necessary for the calculation of these indices when using MSEM with maximum likelihood. While the Posterior Predictive checking approach is available as a model fit index under Bayesian estimation, its suitability for testing isomorphism in MSEMs with ordinal indicators has not yet been explored. A secondary goal of this article is to examine this issue.

**Figure 1:**
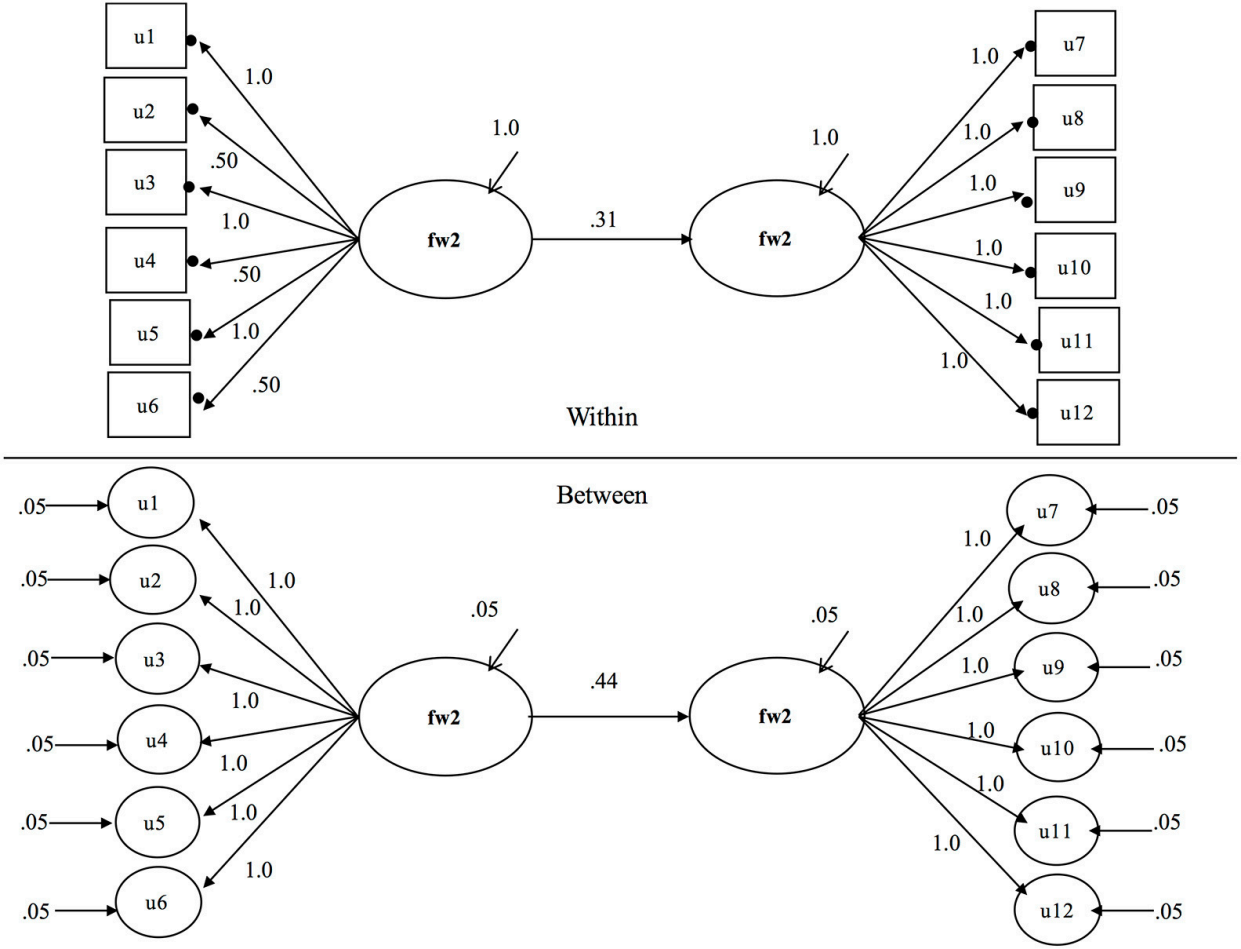
**Least isomorphic and homologous multilevel structural equation model from which all other models can be obtained**.

The model used as the basis of simulations is presented in Figure 1. This model is a two level structural equation model with categorical factor indicators on the within level and random continuous latent indicators on the between level. The solid circles at the ends of the arrows that emanate from the latent within factors fw1 and fw2 indicate random intercepts that vary across clusters. On the between level these are cluster level random intercepts which serve as the indicators of fb1 and fb2. These random intercepts are presented in circles since they are the continuous latent random variables that vary across clusters. For an equation based representation of the parameters of MSEMs with categorical outcomes readers are referred to Grilli and Rampichini (2007) or Jak et al. (2014a). Parameter values used in the Monte Carlo study for this model are included on Figure 1 and are discussed further in the simulation design section below.

### Bayesian estimation of multilevel structural equation models

MSEM involves sampling at both individual and group levels. At level-2, i.e., the group level, samples are often characterized by small numbers of groups, particularly when the population itself is small such as when countries are studied. Modeling of effects at level-2 (or higher) also requires some variation at the higher level (i.e., between groups) as captured by the intraclass correlation for variables. The precise lower limit of the ICC required for modeling group level effects is not firmly established, and sometimes level-2 effects are studied even if ICCs for variables are low indicating little variation between groups. This is especially if the group level effects are of theoretical interest.

However, the combination of low ICCs and low numbers of level-2 units creates problems for maximum likelihood estimation of MSEMs, which assumes large samples and normality. Recent research has shown Bayesian estimators to be more accurate than maximum likelihood and to produce fewer inadmissible solutions with lower numbers of level-2 units (Hox et al., 2012, 2014; Depaoli and Clifton, 2015). As summarized by Depaoli and Clifton (p. 330): “a Bayesian approach to multilevel SEM should produce more accurate and efficient estimates because of shrinkage [toward the mean of the prior]. It should also eliminate problems with convergence due to negative variance estimates because priors can be used to bound estimates to positive values.” In addition to these benefits, Hox et al. (2015) report that Bayesian estimation is more reliable in small samples and is better for complex models.

For these reasons, in the current study we adopted a Bayesian approach to model estimation. Whereas, frequentist estimation methods obtain a single value for parameters, under Bayes estimation a distribution for parameters is obtained. This distribution reflects uncertainty about parameters before data are collected and the likelihood of data that is collected to create the posterior distribution. In Mplus, the software used in the current study, this posterior distribution is sampled using Markov Chain Monte Carlo (MCMC) methods based on the Gibbs Sampler to produce point estimates and confidence intervals. For further details we refer readers to Kruschke (2011), van de Schoot et al. (2014), Zyphur and Oswald (2013), or Lynch (2007) for introductory treatments and to Gelman et al. (2004) for more advanced discussion.

### Hypotheses

Our expectations of the impact of construct isomorphism on construct homology (i.e., equivalence of structural parameters) are as follows. First, when measurement models appropriately model the simulated invariance and simulated non-invariance (i.e., correctly modeled lower loadings on the within levels of models, we anticipate that structural relationships will be accurately recovered according to all model performance statistics we shortly introduce. Second, where measurement models specify loadings on the within and between levels as invariant when in fact they are not (i.e., ignoring non-invariant loadings on the within and between levels in the case of higher between level factors) the direction of regression parameter bias will depend on whether the ignored non-invariance in the measurement model is for the exogenous or the endogenous variable. In the exogenous case, we expect overestimation of the structural coefficient on the between level and underestimation of the structural coefficient on the between level. When the non-invariant measurement model is in the endogenous position, we expect underestimation of the within level structural coefficient and overestimation of the between level structural coefficient.

## Materials and methods

### Fixed features of simulation design

Our design and reporting approach to examine these hypotheses broadly follows the stages discussed by Paxton et al. (2001), Bandalos (2006), and Boomsma et al. (2013).

### Test length and rating scale

We used six items for the independent and dependent latent variable measurement models in the current study. This falls between the three item measurement models reported by Depaoli and Clifton (2015) and eight item models reported by Kim et al. (2012). The reason for adopting six items was that early simulations showed this number was sufficient to illustrate the pattern of the effects of ignored isomorphism on structural coefficients. We used binary indicators for all measurement models, the most discrete response scale possible.

### Number of replications

A review of sample sizes used in recently reported Monte Carlo studies showed that among the largest number of simulations per cell was the study reported by Guenole and Brown (2014) who used 1000 replications per cell of their Monte Carlo design, as did Depaoli and Clifton (2015). We also implemented 1000 replications per cell.

### Types of non-isomorphism

In categorical indicator multilevel models there are no threshold parameters on the within level as the mean and threshold structure is on the between level. The latent mean and thresholds on the between level in this study were set at zero in the population and freely estimated in models. Neither are there any residual variances on the within level, rather, the variances on the within component of the MSEM are fixed at 1 due to the probit link functions used by the estimation software. The only common parameters on within and between levels are the factor variances and loadings. In this study we therefore focus on loading isomorphism.

### Direction of non-isomorphism

Pornprasertmanit et al. (2014) observed standardized loadings are often higher on the between level, and Zyphur et al. (2008) stated “another notable result of the multi-level EFA was the much larger factor loadings found at the between-groups level of analysis, indicating that the between-groups variance may be considered much more reliable than the within-groups variance.” Higher between level loadings are also observable in applied examples of multilevel CFA. For instance, Dyer et al. (2005) presented results of a multilevel CFA of a procedural leadership scale that assessed the extent to which being formal, habitual, cautious, procedural, or ritualistic relate to effective leadership found that loadings were considerably higher on the between level. Hanges and Dickson (2006) showed a similar result for an uncertainty avoidance scale. Accordingly, we study the situation where the non-isomorphism manifests as a higher factor loading on the between level. We note, however, that equal unstandardized loadings or items that are lower on the within level are certainly not impossible and represent cases not covered in the current Monte Carlo design.

### Missing data

We did not simulate missing data in the Monte Carlo results we report below. Missing data can impact conclusions in MSEM, but this issue is beyond the scope of the current study. We refer readers to Hox (2010) or Heck and Thomas (2015) for further discussion of missing data issues in the context of multilevel SEM.

### Experimental conditions

#### Modeling approaches (2 levels)

Two modeling approaches were examined. In the first approach, model parameters on the within and the between level were freely estimated regardless of the fact that the population model specified that they were non-invariant. We did not constrain loading parameters equal for items that were known to be equivalent by design, since equating of probabilistically equivalent items could have confounded conclusions. To also constrain the items that are probabilistically isomorphic to be exactly isomorphic runs the risk of contaminating conclusions by mixing the effects of ignoring the non-isomorphism in items that are non-isomorphic by design with the effects of ignoring probabilistic non-isomorphism due to chance. In the second approach, loadings on the within and between levels for non-isomorphic items were constrained to be equal across levels, regardless of the fact that the population model specified that they were not equivalent.

#### Structural models (4 levels)

Chen et al. (2007) looked at the relationship between leader relationships (LMX), empowerment, and performance. The individual level relationship between LMX and empowerment was 0.31. The relationship between team LMX and team empowerment was 0.44. We adopted these in non-homologous models. In homologous conditions we set the value of the within path at 0.44, the same value as the between level structural path. We included conditions where the non-invariant measurement model occupied the exogenous and endogenous positions. We do not consider non-isomorphic measurement models in exogenous and endogenous positions.

#### Level 1 and level 2 sample sizes (3 levels)

Sample size must be considered at level-1 and level 2. Hox (2010) suggested 10–20 as an appropriate level-1 sample size range. We use one condition of 20 at level-1 because level-1 sample size is rarely a problem in multilevel modeling. Even singletons can be incorporated if the average cluster size is larger and the Level-2 sample size is not too small (Bell et al., 2010). Multilevel Monte Carlo studies by Meuleman and Billiet (2009) and Hox et al. (2012) did not vary level-1 cluster sizes either, albeit they used an imbalanced design to match the cluster sizes reported in European Social Survey data. Maas and Hox (2005) reported minimal detrimental impact on estimator accuracy with even extreme levels of cluster imbalance and hence we only study balanced cluster conditions. More pertinent is the level-2 sample size. Mass and Hox observed sizes as small as 20 can produce accurate estimates of regression parameters, but that if the interest is in the variance parameters then 50 clusters is appropriate for small models and 100 are needed for complex models. We incorporated three level-2 sample sizes of 30, 50, and 100 units.

#### Size of intra-class correlations (ICCs) (4 levels)

At least two approaches have been presented in recent Monte Carlo studies with regard to ICCs. Kim et al. (2012) focused on varying the latent ICC by setting the within factor variance at one and varying the between level factor variance to produce latent ICC values between 0.09 and 0.33. Depaoli and Clifton (2015) varied the ICCs for the observed indicators by fixing the factor loadings at one on the within and between levels and varying the variances and residual variances. We follow Depaoli and Clifton's approach to create observed indicator ICC values of 0.05, 0.10, 0.20, and 0.30.

#### Proportion of non-equivalent items (4 levels)

We incorporated four levels of loading non-isomorphism. These were zero ignored non-isomorphic loadings, one ignored non-isomorphic loading, two ignored non-isomorphic items, and three ignored non-isomorphic items. Our rationale for not going any higher than this is that researchers would be unlikely to be confident that constructs had the same meaning across levels with greater than 50% level of non-invariant loadings unless partial metric isomorphism was the focus of the investigation, and here we focus on strong metric isomorphism.

#### Summary of experimental design

The total number of conditions considered in this Monte Carlo experiment equals 2 modeling approaches × 4 structural models × 3 sample size conditions × 4 ICC conditions × 4° of non-invariance = 384 conditions.

### Analyses

#### Model identification

Parameters for simulation models are illustrated in Figure 1 which presents the least isomorphic and least homologous model studied in the simulation with an intra-class correlation of 0.05 where the non-isomorphic measurement model occupies the exogenous position in the structural model. Remaining models can be reached by making models less isomorphic and homologous according to the specifications in the experimental design section above. To identify the metric of the latent factors we fixed the first factor loading of each factor on within and between levels. This allowed the independent latent variable variances and dependent latent variable residual variances to be freely estimated on both within and between levels in the structural components of the models. This approach was also used to identify multilevel models by Ryu (2014). It is important to note that this approach assumes that the reference indicator must be invariant. In the current article, the referent indicator was indeed invariant, it was so by design. In practice, researchers might consider other approaches. For instance, Jak et al. (2013, 2014a) recommended fixing the within-level factor variance at 1, and freeing the between-level factor variance when factor loadings are constrained to be equal across levels to avoid the risk of picking the “wrong” item for scaling.

#### Estimation

All models were fitted to the simulated item responses in MPlus 7.3 (Muthén and Muthén, 1998–2010). The simulations were executed by calling MPlus from the statistical computing environment R 3.0 using the package MPlusAutomation (Hanges and Dickson, 2006). We use a Bayesian estimator with uninformative priors and a single chain, which closely parallels the set-up reported by Depaoli and Clifton (2015) and Hox et al. (2012), due to the expected superior performance under these conditions. Mplus default settings were used for burn-in while the MCMC process reached target distributions and thinning to reduce dependence in the MCMC draws. The Proportional Scale Reduction (PSR) criterion was used to determine convergence along with visual inspection of trace plots. As we discuss below, we further investigated convergence with runs for extreme calls that incorporated multiple chains and many more iterations. Mplus input and output files for all cells of the design are available at the following link https://dx.doi.org/10.6084/m9.figshare.2069337.v1.

#### Prior specification

Uninformative priors were used since in the small sample conditions an informative prior could overpower the information in the data (Hox et al., 2012). On the within and between levels variance parameter priors were inverse gamma corresponding to a uniform distribution *U*~[0, infinity]. Loading parameter priors on the within and between levels were *N*~(1, 0.1). Threshold priors on the between level were *N*~(0, infinity). Regression mean hyper-parameters were set to the population values with variance of 0.10 so that the estimates would have greatest density in the region of the generating value. For a technical discussion of the details of Bayesian estimation in the context of MSEMs readers are referred to Hox (2010) or Asparouhov and Muthén (2010).

#### Model performance

We examined the following indicators of model performance a) the proportion of non-converged and inadmissible solutions b) the average posterior predictive *p*-value across replications b) the sum of loading errors across items within levels (since these were always in the same direction within conditions and the sum indicated the direction of the bias whereas the absolute bias does not) and c) the relative bias of latent variances and latent residual variances, defined as the observed regression parameter minus the true parameter divided by the true parameter (relative bias of less than 10% was considered acceptable, between 10 and 20% as substantial, and greater than 20% as unacceptable). Finally, while the section of our design that ignored non-isomorphism contained model set ups that were non-isomorphic by design, we also comment on the ability of posterior predictive *p*-values to distinguish correctly and incorrectly specified models reflecting the varying degrees of non-isomorphism in the study.

## Results

### Convergence checks and admissibility

All models in all conditions converged to admissible solutions. This finding is consistent with research by Depaoli and Clifton (2015) that reported that the convergence rates for Bayesian estimation were near 100% even with uninformative priors. The only conditions that these researchers reported did not show 100% convergence and admissibility rates were for combinations of very low ICCs and very small level-2 sample sizes, two conditions that were not incorporated in this simulation study for that reason.

van de Schoot et al. (2015a) observed that variance parameters estimated with Bayesian methods can be subject to spikes (i.e., extreme estimates) especially for variance terms, which inflate parameter estimates. To examine whether this occurred in the current Monte Carlo study we checked trace plots for the within and between exogenous latent variance and latent residual variance parameters for a sample run from each of the 384 cells in the design. These showed that in general the trace plots displayed tight horizontal bands without any obvious increasing or decreasing patterns in the plots. These sample files are uploaded to figshare at the following link: https://dx.doi.org/10.6084/m9.figshare.1619654.v3.

We then examined the issue of convergence further using the following approach. As a first step, we first identified the cells of the Monte Carlo design that had the largest estimate variability for the four latent variance parameters in the models, the within latent exogenous variance, within latent residual variance, and the between latent exogenous variance and between latent residual variance. For the within latent exogenous variance and within latent exogenous residual variance, these cells turned out to be from the correctly specified section of the design, they were cells numbered 100 and 148. These cells had the lowest number of level 2 units and the smallest ICC values (*j* = 30, ICC = 0.05) in the simulation.

These cells showed poor parameter recovery for the regression parameters of either or both within and between level structural coefficients, even though they were correctly specified models. As we explain below, they are excluded from the results presented below because of the impact of ignored non-isomorphism on regression parameter recovery since they were poorly estimated even when correctly specified. In addition to running further checks on the accuracy for these excluded cells, therefore, we also identified the cells with the greatest variation in these parameters that were retained for further investigation of convergence. These cells where cell 100 and cell 148, respectively. Finally, we identified the cells with the largest estimation variability for the between level latent exogenous variance and between level latent residual variance, which were cells 15 and 156, respectively.

For all six specified cells we followed the following steps. First, we re-ran each of these cells using two chains with 100,000 iterations, retaining the PSR criterion, and we requested Kolmogorov-Smirnov tests using the Tech 9 option in Mplus and confirmed there were no significant results. In addition to these precautions, trace plots for a random run from each of these conditions inspected to ensure visual inspection of the plots showed good mixing and no obvious spiking. Results of these analyses for cells 100 and 148 showed that the structural regression parameters were still too poorly estimated to warrant inclusion in the comparison of the isomorphism misspecification just as was the case when using the default convergence criteria.

The estimates of the within latent exogenous variance and within latent residual variance in these cells were extremely close to the estimates from letting Mplus converge based on the program's default criteria. For the within latent exogenous variance, the population value was 1.000, the default criteria converged to 0.962 and the longer run with additional diagnostics converged to 0.956. For the within latent residual variance these values were 1.000, 1.086, and 1.065.

For the within latent exogenous variance in cell 16 the population variance was 1.000, the Mplus default convergence criteria produced 0.970, while the longer run with additional diagnostics converged to 0.963. For cell 156, the retained cell with the greatest estimate variability for the within latent residual variance, the population parameter was 1.000, the estimate produced by Mplus defaults was 1.053, and the estimate produced by the longer run with greater iterations and the additional convergence checks was 1.059.

Next we examined the cells with the greatest estimate variability for the between latent exogenous variance and between latent residual variance, cells 303 and 256, respectively. For the between latent exogenous variance the population value was 0.44, the estimate produced by Mplus default convergence criteria was 0.614 and the estimate produced by the greater iterations and multiple convergence criteria was 0.609. The corresponding values for the between latent residual variance were 0.44, 0.674, and 0.664.

Given that these checks on the cells with the largest variability in estimates show that the default Mplus convergence criteria lead to very similar estimates to much longer iterations in the context of this study, we concluded that the other cells in the design which showed less variability in estimates of these parameters have very likely converged too under the default convergence criteria used by the Mplus software.

### Results of posterior predictive checking

Posterior predictive *p*-values were examined as an indicator of global fit for all models. The first results we consider are for the correctly specified conditions. It was important to incorporate this condition because if we did not, it would be impossible to know to what extent parameter bias in the misspecified condition was due to poor estimation and how much was due to the misspecification under study. In other words, we needed to ensure that the correctly specified models were estimated accurately so that any error in the models for the misspecified condition could clearly be attributed to the ignored non-isomorphism. The *ppp*-values for the correctly specified conditions showed that the minimum proportion of replications in each cell with a *PPP* > 0.50 was 0.45. For this reason, we do not discuss global fit or parameter estimation accuracy for the correctly specified conditions further in this article. However, a zip file of all Mplus input and output files, as well as a summary excel file where all parameters are extracted for convenient reading, are available at the following link https://dx.doi.org/10.6084/m9.figshare.2069334.v1.

The ppp statistic is known to be insensitive to small model misspecifications with categorical data (Asparouhov and Muthén, 2010). Our results confirmed this finding. Moving from the correctly specified models through with zero items with ignored non-invariance through to three items with ignored non-invariance did not result in deterioration in the *ppp*-values, suggesting that the *ppp*-value is insensitive to the degrees of ignored isomorphism examined in this design, even for many ignored non-isomorphic items, large intra-class correlations, and the highest number of level-2 units. While examining the efficacy of the *ppp*-value to test isomorphism was not the central focus of this article, it is nevertheless an important topic and we discuss future research directions for testing isomorphism in multilevel modeling with Bayesian estimators in our Discussion section.

### Local accuracy results for correctly specified models

All results we now discuss are available at the following link https://dx.doi.org/10.6084/m9.figshare.2069334.v1. We examined local fit in terms of the relative bias of the regression parameters on the within and between levels, as the regression coefficients summarized the key result we are focusing on in this study. The relative bias for the regression parameters on the within and between levels for the correctly specified conditions were always acceptable on average with a single exception. The only conditions where this pattern was violated was for the lowest level of ICC = 0.05. In this condition, even the largest level 2 sample size of 100 was not enough to offset the effect of small ICCs on estimation accuracy, despite that level-2 sample size and ICC values are known to have an interactive impact on accuracy in the context of multilevel models.

In the current study, increasing the sample size from 30 to 50 and 100 while maintaining the 0.05 ICC for the latent variable ameliorated, but did not eliminate the estimation error. For this reason, in the results that follow for the misspecified condition, we remove conditions where the ICC was 0.05, since any estimation error in the incorrectly specified model would not be uniquely attributable to the intended model misspecification. Estimation in these other conditions was deemed acceptable and we therefore do report these conditions were the items were misspecified as being invariant. We do not discuss the parameter accuracy of the correctly specified conditions further in this article, however, these results can be examined in both the original Mplus input and output scripts and excel summary file mentioned earlier.

### Local accuracy results for misspecified model conditions

All results we now discuss are available at the following link https://dx.doi.org/10.6084/m9.figshare.2069334.v1. We now turn to discussion of local estimation accuracy, beginning with discussion of the within and between loadings and latent variance and residual latent variances, before turning to within and between regression coefficients. The tables of results for each of these sets of parameters are presented along with discussion of results below. Tables 1, 2 below present an overall summary of trends in estimation error of regression coefficients, variances, and loadings on within and between levels. Each cell of this table contains two pieces of information. First the table lists the sign of the misestimation of the specific parameter for the highest degree of ignored non-isomorphism. These values always take on one of the following three values: negative, acceptable, or positive. Second, each cell also contains the direction of change in the estimation error due to increasing levels of ignored non-isomorphism. These cells entries read either yes or no. A yes indicates that increased ignored non-isomorphism led to increased bias in the specified direction, while no indicates that there was little or no change in the estimation accuracy from the lowest to the highest degree of ignored non-invariance.

**Table 1:** **Summary of impact of ignored isomorphism on structural coefficients**.

	**Misspecified IV**		**Misspecified DV**	
Within beta	Positive	Yes	Negative	Yes
Between beta	Negative	Yes	Positive	Yes

*Each cell of this table contains two pieces of information, the sign of the misestimation of for the parameter under the highest degree of ignored non-isomorphism (negative, acceptable, or positive), and the direction of change in the estimation error due to increasing levels of ignored non-isomorphism where yes indicates that increased ignored non-isomorphism led to increased bias in the specified direction and no indicates that there was little or no change in the estimation accuracy from the lowest to the highest degree of ignored non-invariance*.

**Table 2:** **Summary of impact of ignored isomorphism on loadings and variances**.

	**Misspecified IV**		**Misspecified DV**	
	**Latent IV**	**Latent DV**	**Latent IV**	**Latent DV**
Within loading	Negative	Negative	Negative	Negative
	Yes	No	Yes	No
Between loading	Positive	Acceptable	Positive	Acceptable
	Yes		Yes
Within variance/Residual variance	Negative	Positive	Negative	Negative
	Yes	No	Yes	Yes
Between variance/Residual variance.	Positive	Acceptable	Acceptable	Positive
	Yes			Yes

*Each cell of this table contains two pieces of information, the sign of the misestimation of for the parameter under the highest degree of ignored non-isomorphism (negative, acceptable, or positive), and the direction of change in the estimation error due to increasing levels of ignored non-isomorphism where yes indicates that increased ignored non-isomorphism led to increased bias in the specified direction and no indicates that there was little or no change in the estimation accuracy from the lowest to the highest degree of ignored non-invariance*.

#### Within and between loadings

Tables 3–6 contain the sum of bias across factor loadings for conditions where the non-isoporphism was on the exogenous and endogenous measurement models and when the structural coefficient was homologous and non-homologous. Within the exogenous and endogenous conditions the bias for loadings was always in the same direction, and so here we report the sum of bias. Given that the bias was generally very small loadings about zero or positive (or negative) within conditions this is similar to reporting the absolute bias except the sign of the bias is maintained. When the misspecified measurement model was in the exogenous position the exogenous loading bias was negative and the bias increased with greater ignored non-isomorphism. On the between level the exogenous loading bias was positive and the positive bias increased with greater ignored non-isomorphism. The endogenous latent loadings were negative and stable with increased non-invariance on the within level and were acceptable on the between level. When the misspecified measurement model was in the endogenous position the exogenous latent loadings on the within level were negatively biased and the bias increased with further un-modeled non-isomorphism while the loading bias on the between level was positive and increasingly so with greater non-isomorphism. The loading bias for the endogenous latent variable was negative and stable with further non-isomorphism while the loading bias on the between level for the endogenous latent was acceptable.

**Table 3:** **Latent variable parameter accuracy for homologous condition with non-invariant IV measurement models**.

***j***	**ICC**	**Items**	**Within Latent IV**			**Within Latent DV**			**Between Latent IV**			**Between Latent DV**		
			**Load Sum Bias**	**Var Rel Bias (%)**	**Var Cov**	**Load Sum Bias**	**Var Rel Bias (%)**	**Var Cov**	**Load Sum Bias**	**Var Rel Bias (%)**	**Var Cov**	**Load Sum Bias**	**Var Rel Bias (%)**	**Var Cov**
30	0.10	0	−0.38	1	0.92	−0.18	8	0.89	−0.36	−3	0.73	0.10	4	0.89
		1	−0.55	−6	0.87	−0.19	7	0.90	0.73	35	0.86	0.02	13	0.91
		2	−0.75	−13	0.80	−0.18	7	0.91	1.20	58	0.88	0.06	10	0.90
		3	−0.94	−21	0.70	−0.19	7	0.90	1.40	72	0.84	0.07	10	0.91
	0.15	0	−0.39	0	0.89	−0.18	8	0.89	−0.44	1	0.86	0.20	6	0.94
		1	−0.70	−10	0.81	−0.21	7	0.89	0.62	32	0.95	0.21	4	0.93
		2	−1.05	−21	0.67	−0.18	7	0.91	1.02	52	0.87	0.10	5	0.93
		3	−1.31	−31	0.54	−0.21	6	0.90	1.15	59	0.83	0.08	9	0.93
	0.20	0	−0.39	0	0.90	−0.22	6	0.90	−0.37	3	0.93	0.22	5	0.95
		1	−0.90	−15	0.76	−0.19	7	0.90	0.50	27	0.93	0.16	6	0.94
		2	−1.32	−28	0.57	−0.19	8	0.90	0.83	41	0.89	0.20	6	0.94
		3	−1.66	−39	0.40	−0.17	8	0.90	0.89	46	0.87	0.18	7	0.93
50	0.10	0	−0.19	1	0.91	−0.08	5	0.92	−0.24	2	0.84	0.07	5	0.94
		1	−0.40	−6	0.87	−0.11	5	0.91	0.80	34	0.92	0.06	7	0.91
		2	−0.42	−8	0.87	−0.09	5	0.91	0.72	25	0.93	0.09	5	0.93
		3	−0.84	−23	0.62	−0.12	3	0.91	1.47	75	0.68	0.06	7	0.91
	0.15	0	−0.19	0	0.92	−0.10	4	0.94	−0.20	1	0.91	0.16	3	0.93
		1	−0.61	−12	0.79	−0.09	5	0.91	0.72	31	0.91	0.13	4	0.93
		2	−0.95	−23	0.59	−0.10	4	0.91	1.09	51	0.80	0.07	6	0.94
		3	−1.23	−33	0.40	−0.07	5	0.92	1.19	56	0.75	0.05	6	0.93
	0.20	0	−0.22	0	0.89	−0.10	5	0.91	−0.23	1	0.93	0.12	5	0.93
		1	−0.79	−17	0.73	−0.12	4	0.91	0.59	26	0.90	0.14	2	0.94
		2	−1.25	−31	0.43	−0.11	4	0.92	0.88	39	0.83	0.11	5	0.95
		3	−1.66	−43	0.20	−0.12	4	0.90	0.87	40	0.84	0.12	3	0.95
100	0.10	0	−0.09	0	0.94	−0.09	1	0.91	−0.15	0	0.89	0.12	1	0.91
		1	−0.33	−8	0.84	−0.04	3	0.91	0.89	37	0.86	0.06	2	0.92
		2	−0.33	−9	0.83	−0.03	3	0.91	0.81	24	0.91	0.02	4	0.93
		3	−0.78	−24	0.44	−0.05	2	0.92	1.52	69	0.43	0.04	4	0.93
	0.15	0	−0.11	0	0.91	−0.05	2	0.91	−0.17	0	0.92	0.07	1	0.94
		1	−0.47	−12	0.78	−0.04	3	0.92	0.80	30	0.84	0.06	2	0.93
		2	−0.90	−25	0.41	−0.05	2	0.90	1.13	48	0.62	0.05	2	0.95
		3	−1.17	−35	0.18	−0.07	2	0.91	1.22	53	0.50	0.05	2	0.94
	0.20	0	−0.10	0	0.91	−0.05	2	0.92	−0.08	2	0.94	0.04	2	0.93
		1	−0.68	−17	0.62	−0.05	2	0.91	0.67	26	0.85	0.06	1	0.94
		2	−1.26	−34	0.12	−0.06	1	0.92	0.87	36	0.70	0.07	1	0.93
		3	−1.61	−44	0.04	−0.05	2	0.92	0.89	37	0.68	0.06	2	0.94

*j is the number of level-2 units; ICC, intra-class correlation; items refers to the number of ignored non-isomorphic items; IV, Independent variable; DV, dependent variable; Load, Loading; Sum Bias, sum of absolute bias on loading parameters; Var, latent factor variance if it is under a latent IV heading and latent residual variance if it is under a latent DV heading; Rel Bias, relative bias expressed as a percentage; Cov, coverage expressed as a proportion*.

**Table 4:** **Latent variable parameter accuracy for homologous condition with non-invariant DV measurement model**.

***j***	**ICC**	**Items**	**Within Latent IV**			**Within Latent DV**			**Between Latent IV**			**Between Latent DV**		
			**Load Sum Bias**	**Var Rel Bias (%)**	**Var Cov**	**Load Sum Bias**	**Var Rel Bias (%)**	**Var Cov**	**Load Sum Bias**	**Var Rel Bias (%)**	**Var Cov**	**Load Sum Bias**	**Var Rel Bias (%)**	**Var Cov**
30	0.10	0	−0.21	3	0.90	−0.33	7	0.89	−0.10	−1	0.74	0.34	9	0.92
		1	−0.41	1	0.92	−0.36	1	0.89	0.84	2	0.76	0.28	8	0.94
		2	−0.59	2	0.89	−0.32	−7	0.86	1.31	2	0.76	0.14	8	0.90
		3	−0.77	1	0.68	−0.39	−14	0.89	1.42	0	0.76	0.05	76	0.86
	0.15	0	−0.20	0	0.91	−0.36	7	0.89	−0.20	4	0.88	0.38	8	0.94
		1	−0.57	1	0.91	−0.35	−5	0.87	0.73	3	0.86	0.34	36	0.92
		2	−0.85	1	0.88	−0.35	−15	0.77	1.15	−1	0.87	0.37	52	0.86
		3	−1.12	1	0.90	−0.34	−26	0.67	1.29	3	0.87	0.40	69	0.76
	0.20	0	−0.20	0	0.90	−0.37	7	0.92	−0.22	2	0.95	0.42	3	0.94
		1	−0.69	1	0.91	−0.35	−10	0.85	0.61	3	0.92	0.36	30	0.89
		2	−1.16	−1	0.88	−0.39	−23	0.69	0.96	0	0.91	0.43	34	0.93
		3	−1.46	1	0.89	−0.34	−33	0.57	1.03	1	0.92	0.35	53	0.79
50	0.10	0	−0.17	2	0.91	−0.22	2	0.87	−0.12	5	0.84	0.17	0	0.88
		1	−0.29	0	0.90	−0.23	−4	0.92	0.98	−8	0.78	0.32	47	0.87
		2	−0.56	1	0.91	−0.15	−12	0.71	1.35	11	0.85	0.18	54	0.81
		3	−0.72	2	0.93	−0.18	−18	0.73	1.58	3	0.83	0.16	77	0.69
	0.15	0	−0.09	1	0.92	−0.19	6	0.92	−0.11	2	0.90	0.26	3	0.94
		1	−0.52	1	0.92	−0.20	−9	0.83	0.77	3	0.91	0.16	33	0.88
		2	−0.85	1	0.91	−0.17	−20	0.69	1.15	4	0.91	0.24	50	0.78
		3	−1.06	1	0.89	−0.18	−29	0.52	1.30	1	0.89	0.23	63	0.67
	0.20	0	−0.11	1	0.90	−0.21	4	0.90	−0.15	1	0.93	0.22	4	0.93
		1	−0.70	1	0.90	−0.21	−14	0.80	0.65	5	0.93	0.17	29	0.88
		2	−1.17	0	0.91	−0.21	−28	0.53	0.95	1	0.92	0.25	42	0.78
		3	−1.47	1	0.91	−0.18	−38	0.32	1.00	0	0.91	0.23	46	0.74
100	0.10	0	−0.06	0	0.90	−0.10	2	0.91	−0.07	−1	0.89	0.17	3	0.94
		1	−0.29	0	0.90	−0.09	−6	0.85	0.91	1	0.88	0.15	35	0.83
		2	−0.53	0	0.91	−0.10	−15	0.70	1.37	3	0.88	0.09	59	0.57
		3	−0.74	0	0.92	−0.10	−23	0.49	1.54	2	0.90	0.09	70	0.39
	0.15	0	−0.06	0	0.92	−0.11	2	0.93	−0.04	2	0.92	0.08	3	0.94
		1	−0.43	1	0.92	−0.11	−10	0.78	0.81	1	0.90	0.15	30	0.80
		2	−0.30	1	0.92	−0.09	−23	0.49	1.17	2	0.92	0.12	48	0.57
		3	−1.14	1	0.92	−0.09	−34	0.21	1.24	1	0.93	0.10	55	0.45
	0.20	0	−0.04	1	0.90	−0.09	3	0.90	−0.07	1	0.94	0.12	1	0.93
		1	−0.61	0	0.91	−0.10	−16	0.68	0.71	2	0.93	0.10	26	0.82
		2	−1.19	1	0.91	−0.08	−32	0.23	0.93	3	0.93	0.08	37	0.67
		3	−1.55	0	0.90	−0.10	−43	0.05	0.94	1	0.93	0.09	112	0.64

*j is the number of level-2 units; ICC, intra-class correlation; items refers to the number of ignored non-isomorphic items; IV, Independent variable; DV, dependent variable; Load, Loading; Sum Bias, sum of absolute bias on loading parameters; Var, latent factor variance if it is under a latent IV heading and latent residual variance if it is under a latent DV heading; Rel Bias, relative bias expressed as a percentage; Cov, coverage expressed as a proportion*.

**Table 5:** **Latent variable parameter accuracy for non-homologous condition with non-invariant IV measurement model**.

***j***	**ICC**	**Items**	**Within Latent IV**			**Within Latent DV**			**Between Latent IV**			**Between Latent DV**		
			**Load Sum Bias**	**Var Rel Bias (%)**	**Var Cov**	**Load Sum Bias**	**Var Rel Bias (%)**	**Var Cov**	**Load Sum Bias**	**Var Rel Bias (%)**	**Var Cov**	**Load Sum Bias**	**Var Rel Bias (%)**	**Var Cov**
30	0.10	0	−0.35	3	0.91	−0.20	8	0.91	−0.36	−4	0.74	−0.17	5	0.91
		1	−0.59	−6	0.85	−0.18	7	0.91	0.63	32	0.88	−0.09	6	0.93
		2	−0.76	−13	0.80	−0.20	6	0.90	1.19	63	0.89	0.02	11	0.92
		3	−0.96	−23	0.70	−0.20	5	0.90	1.38	74	0.83	0.06	13	0.91
	0.15	0	−0.35	0	0.91	−0.19	8	0.92	−0.40	3	0.85	−0.20	4	0.95
		1	−0.73	−11	0.81	−0.22	5	0.92	0.62	32	0.92	−0.17	8	0.94
		2	−1.02	−21	0.69	−0.18	7	0.89	1.04	50	0.89	−0.09	10	0.94
		3	−1.32	−31	0.53	−0.18	7	0.91	1.13	59	0.86	−0.15	8	0.94
	0.20	0	−0.35	2	0.91	−0.23	6	0.90	−0.38	5	0.90	−0.23	7	0.95
		1	−0.92	−16	0.77	−0.23	6	0.91	0.52	31	0.92	−0.19	5	0.95
		2	−1.39	−29	0.57	−0.18	8	0.91	0.81	−11	0.94	−0.17	40	0.89
		3	−1.68	−39	0.40	−0.24	5	0.88	0.86	44	0.88	−0.21	4	0.95
50	0.10	0	−0.22	0	0.91	−0.13	4	0.90	−0.21	3	0.84	−0.09	6	0.93
		1	−0.44	−7	0.83	−0.12	3	0.91	0.77	35	0.90	−0.02	6	0.92
		2	−0.67	−15	0.72	−0.10	5	0.90	1.28	60	0.82	−0.06	7	0.92
		3	−0.90	−25	0.59	−0.09	0	1.00	1.43	74	0.69	−0.03	7	0.91
	0.15	0	−0.23	−3	0.92	−0.12	4	0.92	−0.26	1	0.90	−0.15	2	0.95
		1	−0.60	−12	0.79	−0.12	4	0.92	0.72	32	0.90	−0.12	4	0.93
		2	−0.96	−23	0.59	−0.10	5	0.91	1.09	50	0.80	−0.05	6	0.94
		3	−1.23	−33	0.40	−0.11	4	0.89	1.19	58	0.74	−0.15	3	0.94
	0.20	0	−0.21	0	0.90	−0.11	5	0.91	−0.20	1	0.92	−0.14	5	0.94
		1	−0.78	−17	0.72	−0.12	4	0.91	0.61	27	0.89	−0.13	4	0.93
		2	−1.32	−32	0.42	−0.13	4	0.91	0.85	38	0.83	−0.16	1	0.94
		3	−1.68	−43	0.21	−0.13	3	0.92	0.84	41	0.83	−0.16	2	0.94
100	0.10	0	−0.08	1	0.94	−0.06	2	0.93	−0.14	1	0.89	−0.10	2	0.90
		1	−0.34	−8	0.84	−0.05	1	0.92	0.86	35	0.86	−0.02	4	0.93
		2	−0.60	−17	0.67	−0.03	3	0.92	1.32	59	0.60	−0.02	4	0.93
		3	−0.80	−25	0.40	−0.06	2	0.92	1.48	69	0.45	0.01	7	0.95
	0.15	0	−0.10	1	0.91	−0.06	1	0.91	−0.12	3	0.89	−0.05	3	0.94
		1	−0.37	−9	0.81	−0.06	2	0.92	0.87	33	0.86	−0.04	4	0.93
		2	−0.58	−16	0.67	−0.06	1	0.93	1.32	54	0.64	−0.03	3	0.92
		3	−0.79	−24	0.45	−0.04	2	0.92	1.49	68	0.44	−0.01	4	0.93
	0.20	0	−0.10	1	0.93	−0.07	1	0.91	−0.10	2	0.92	−0.07	2	0.94
		1	−0.72	−19	0.59	−0.06	1	0.92	0.64	24	0.84	−0.05	3	0.94
		2	−1.25	−34	0.17	−0.05	2	0.91	0.89	37	0.70	−0.08	2	0.94
		3	−1.65	−45	0.03	−0.05	2	0.92	0.87	37	0.71	−0.05	2	0.94

*j is the number of level-2 units; ICC, intra-class correlation; items refers to the number of ignored non-isomorphic items; IV, Independent variable; DV, dependent variable; Load, Loading; Sum Bias, sum of absolute bias on loading parameters; Var, latent factor variance if it is under a latent IV heading and latent residual variance if it is under a latent DV heading; Rel Bias, relative bias expressed as a percentage; Cov, coverage expressed as a proportion*.

**Table 6:** **Latent variable parameter accuracy for non-homologous condition with non-invariant DV measurement model**.

***j***	**ICC**	**Items**	**Within Latent IV**			**Within Latent DV**			**Between Latent IV**			**Between Latent DV**		
			**Load Sum Bias**	**Var Rel Bias (%)**	**Var Cov**	**Load Sum Bias**	**Var Rel Bias (%)**	**Var Cov**	**Load Sum Bias**	**Var Rel Bias (%)**	**Var Cov**	**Load Sum Bias**	**Var Rel Bias (%)**	**Var Cov**
30	0.10	0	−0.20	1	0.90	−0.36	7	0.92	−0.16	−1	0.76	−0.30	3	0.89
		1	−0.45	2	0.91	−0.36	−2	0.85	−0.76	−1	0.75	−0.33	33	0.96
		2	−0.70	1	0.88	−0.35	−11	0.82	1.28	2	0.74	−0.13	58	0.91
		3	−0.82	1	0.91	−0.38	1	0.91	1.49	3	0.76	−0.22	79	0.87
	0.15	0	−0.23	1	0.91	−0.38	7	0.90	−0.19	2	0.87	−0.42	8	0.93
		1	−0.61	0	0.90	−0.38	−7	0.87	0.71	4	0.87	−0.41	33	0.93
		2	−0.89	2	0.91	−0.32	−16	0.80	1.13	4	0.88	−0.34	53	0.83
		3	−1.14	1	0.89	−0.32	−25	0.67	1.25	3	0.89	−0.36	65	0.80
	0.20	0	−0.22	1	0.90	−0.36	6	0.92	−0.26	5	0.96	−0.39	2	0.92
		1	−0.71	0	0.91	−0.38	−10	0.83	0.61	1	0.90	−0.37	29	0.91
		2	−1.28	1	0.90	−0.35	−27	0.64	0.86	2	0.89	−0.43	42	0.86
		3	−1.51	0	0.90	−0.37	−34	0.52	0.98	2	0.92	−0.34	48	0.82
50	0.10	0	−0.15	1	0.90	−0.21	3	0.93	−0.14	2	0.85	−0.30	5	0.93
		1	−0.38	1	0.91	−0.19	−5	0.87	0.84	0	0.84	−0.27	35	0.91
		2	−0.59	1	0.91	−0.20	−13	0.81	1.32	2	0.85	−0.28	59	0.81
		3	−0.81	1	0.91	−0.19	−21	0.68	1.49	5	0.84	−0.24	70	0.73
	0.15	0	−0.14	1	0.90	−0.19	3	0.92	−0.12	4	0.90	−0.22	4	0.94
		1	−0.53	2	0.90	−0.19	−9	0.82	0.75	6	0.92	−0.19	32	0.90
		2	−0.91	1	0.90	−0.19	−22	0.65	1.10	2	0.90	−0.23	52	0.77
		3	−1.19	1	0.91	−0.17	−31	0.48	1.21	3	0.90	−0.21	57	0.73
	0.20	0	−0.11	1	0.94	−0.21	5	0.91	−0.13	0	0.92	−0.26	4	0.95
		1	−0.76	1	0.91	−0.20	−15	0.76	0.62	2	0.94	−0.16	25	0.88
		2	−1.27	0	0.90	−0.22	−30	0.47	0.88	3	0.92	−0.23	40	0.81
		3	−1.64	0	0.91	−0.22	−42	0.28	0.90	3	0.93	−0.20	43	0.77
100	0.10	0	−0.06	1	0.91	−0.09	2	0.92	−0.08	1	0.90	−0.13	4	0.94
		1	−0.33	1	0.92	−0.10	−8	0.84	0.90	3	0.89	−0.11	32	0.84
		2	−0.57	0	0.91	−0.11	−16	0.67	1.34	3	0.87	−0.16	59	0.60
		3	−0.80	0	0.90	−0.10	−25	0.45	1.48	4	0.90	−0.08	67	0.44
	0.15	0	−0.05	1	0.90	−0.08	2	0.92	−0.07	1	0.91	−0.17	2	0.93
		1	−0.33	0	0.92	−0.08	−7	0.83	0.89	2	0.89	−0.14	33	0.86
		2	−0.56	1	0.90	−0.08	−15	0.68	1.33	2	0.89	−0.12	55	0.61
		3	−0.83	0	0.91	−0.10	−25	0.43	1.46	3	0.91	−0.11	66	0.48
	0.20	0	−0.05	2	0.91	−0.10	0	0.88	−0.06	0	0.94	−0.12	1	0.93
		1	−0.69	1	0.91	−0.09	−17	0.63	0.67	3	0.94	−0.08	25	0.83
		2	−1.29	1	0.93	−0.09	−34	0.21	0.86	0	0.94	−0.09	35	0.69
		3	−1.66	0	0.91	−0.09	−45	0.03	0.85	2	0.94	−0.10	35	0.67

*j is the number of level-2 units; ICC, intra-class correlation; items refers to the number of ignored non-isomorphic items; IV, Independent variable; DV, dependent variable; Load, Loading; Sum Bias, sum of absolute bias on loading parameters; Var, latent factor variance if it is under a latent IV heading and latent residual variance if it is under a latent DV heading; Rel Bias, relative bias expressed as a percentage; Cov, coverage expressed as a proportion*.

#### Within and between latent variances and latent residual variances

Tables 3–6 present the relative bias for the latent variances and latent residual variances. These tables show that when the non-invariant measurement model occupied an exogenous position in the structural model, the relative bias in exogenous latent variance on the within level was negative and this negative bias increased with increased ignored non-isomorphism, while the between level exogenous latent variance was positive and the positive bias increased with higher levels of ignored non-isomorphism. The endogenous latent residual variance on the within level was negatively biased, but was stable with increased ignored non-isomorphism. On the between level the latent residual variance relative bias was acceptable. When the misspecified measurement model was in the endogenous position the within level relative bias was negative and increasingly so with further ignored non-isomorphism. On the between level the latent variance relative bias was acceptable. The residual latent variable variance on the within level was increasingly negative with greater ignored non-isomorphism while the between level residual variance was positively bias, with bias increasing as more non-isomorphism was ignored.

#### Within and between structural coefficients

Tables 7–10 below summarize the accuracy for structural coefficients on the within and between level where the non-invariant measurement model was in the exogenous and endogenous position in the structural model and when the structural relationship was homologous and non-homologous. This table reveals consistent patterns across simplify reporting of the results. First, increased levels of misspecification consistently led to increased relative bias. Unacceptable increases in relative bias occurred for even a single equated but non-isomorphic item. When the ignored non-isomorphism was on the exogenous measurement model, the within level structural coefficient became increasingly positively biased and the between level structural coefficient became increasingly negatively biased with increased ignored non-isomorphism. When the ignored non-isomorphism was on the endogenous measurement model the within level structural coefficient became increasingly negatively biased and the between level structural coefficient became increasingly positively biased with greater ignored non-isomorphism.

**Table 7:** **Structural parameter accuracy for homologous condition with non-invariant IV measurement model**.

***j***	**ICC**	**Items**	**Within Beta**		**Between Beta**	
			**Rel bias (%)**	**Coverage**	**Rel bias (%)**	**Coverage**
30	0.10	0	5	0.93	7	0.96
		1	8	0.91	−15	0.95
		2	15	0.90	−17	0.91
		3	20	0.87	−21	0.89
	0.15	0	7	0.91	7	0.95
		1	10	0.90	−10	0.95
		2	20	0.86	−19	0.92
		3	28	0.82	−17	0.92
	0.20	0	5	0.93	2	0.95
		1	15	0.89	−9	0.95
		2	24	0.84	−12	0.93
		3	35	0.77	−15	0.92
50	0.10	0	4	0.94	−1	0.95
		1	6	0.93	−11	0.93
		2	9	0.91	−11	0.93
		3	18	0.83	−23	0.86
	0.15	0	3	0.94	5	0.95
		1	10	0.89	−12	0.96
		2	18	0.83	−16	0.90
		3	28	0.74	−17	0.90
	0.20	0	3	0.93	4	0.96
		1	13	0.88	−10	0.94
		2	24	0.78	−16	0.93
		3	37	0.63	−15	0.92
100	0.10	0	1	0.93	1	0.93
		1	6	0.90	−15	0.90
		2	7	0.90	−12	0.91
		3	17	0.74	−24	0.82
	0.15	0	1	0.93	1	0.95
		1	8	0.89	−11	0.92
		2	18	0.73	−17	0.89
		3	25	0.61	−19	0.85
	0.20	0	2	0.91	2	0.94
		1	11	0.83	−9	0.93
		2	24	0.62	−14	0.92
		3	37	0.37	−14	0.90

*j is the number of level-2 units; ICC, intra-class correlation; items refers to the number of ignored non-isomorphic items; Rel bias, relative bias expressed as a percentage; Cov, coverage expressed as a proportion*.

**Table 8:** **Structural parameter accuracy for homologous condition with non-invariant DV measurement model**.

***j***	**ICC**	**Items**	**Within Beta**		**Between Beta**	
			**Rel bias (%)**	**Coverage**	**Rel bias (%)**	**Coverage**
30	0.10	0	3	0.94	3	0.97
		1	0	0.92	10	0.95
		2	−3	0.92	31	0.94
		3	−7	0.87	38	0.94
	0.15	0	5	0.92	1	0.96
		1	−1	0.93	19	0.95
		2	−7	0.89	23	0.95
		3	−13	0.86	33	0.94
	0.20	0	6	0.94	2	0.95
		1	−4	0.91	14	0.96
		2	−12	0.85	34	0.93
		3	−18	0.80	29	0.94
50	0.10	0	4	0.90	17	0.88
		1	3	0.96	27	0.92
		2	−4	0.91	26	0.90
		3	−10	0.87	36	0.91
	0.15	0	3	0.92	4	0.95
		1	−4	0.91	13	0.95
		2	−11	0.85	25	0.93
		3	−16	0.78	33	0.93
	0.20	0	2	0.92	2	0.96
		1	−7	0.95	13	0.95
		2	−15	0.78	22	0.93
		3	−22	0.67	26	0.94
100	0.10	0	1	0.94	2	0.95
		1	−3	0.92	16	0.94
		2	−8	0.86	28	0.89
		3	−13	0.75	33	0.88
	0.15	0	1	0.93	0	0.95
		1	−5	0.89	16	0.94
		2	−12	0.78	21	0.90
		3	−19	0.56	27	0.89
	0.20	0	2	0.92	1	0.94
		1	−8	0.86	11	0.95
		2	−18	0.61	18	0.92
		3	−24	0.38	20	0.91

*j is the number of level-2 units; ICC, intra-class correlation; items refers to the number of ignored non-isomorphic items; Rel bias, relative bias expressed as a percentage; Cov, coverage expressed as a proportion*.

**Table 9:** **Structural parameter accuracy for non-homologous condition with non-invariant IV measurement model**.

***j***	**ICC**	**Items**	**Within Beta**		**Between Beta**	
			**Rel bias (%)**	**Coverage**	**Rel bias (%)**	**Coverage**
30	0.10	0	4	0.94	15	0.96
		1	9	0.93	−8	0.95
		2	14	0.91	−22	0.92
		3	20	0.90	−19	0.90
	0.15	0	6	0.93	3	0.96
		1	12	0.93	−8	0.95
		2	16	0.92	−10	0.93
		3	29	0.86	−17	0.92
	0.20	0	2	0.92	5	0.96
		1	13	0.92	−5	0.96
		2	25	0.88	−11	0.94
		3	34	0.84	−14	0.93
50	0.10	0	3	0.93	8	0.94
		1	7	0.93	−12	0.94
		2	13	0.92	−17	0.88
		3	20	0.88	−18	0.88
	0.15	0	2	0.94	3	0.93
		1	9	0.93	−10	0.95
		2	19	0.88	−15	0.92
		3	27	0.81	−19	0.91
	0.20	0	4	0.94	3	0.94
		1	13	0.88	−7	0.95
		2	25	0.83	−14	0.92
		3	36	0.75	−13	0.92
100	0.10	0	1	0.94	2	0.95
		1	6	0.90	−12	0.93
		2	12	0.88	−19	0.87
		3	17	0.82	−19	0.85
	0.15	0	1	0.94	2	0.95
		1	6	0.93	−12	0.92
		2	11	0.88	−18	0.88
		3	17	0.81	−21	0.84
	0.20	0	1	0.93	1	0.95
		1	12	0.87	−9	0.94
		2	25	0.67	−13	0.89
		3	37	0.53	−13	0.91

*j is the number of level-2 units; ICC, intra-class correlation; items refers to the number of ignored non-isomorphic items; Rel bias, relative bias expressed as a percentage; Cov, coverage expressed as a proportion*.

**Table 10:** **Structural parameter accuracy for non-homologous condition with non-invariant DV measurement model**.

***j***	**ICC**	**Items**	**Within Beta**		**Between Beta**	
			**Rel bias (%)**	**Coverage**	**Rel bias (%)**	**Coverage**
30	0.10	0	5	0.93	9	0.97
		1	−1	0.91	25	0.95
		2	−5	0.91	31	0.95
		3	−8	0.90	46	0.94
	0.15	0	5	0.93	5	0.96
		1	−4	0.94	20	0.93
		2	−9	0.92	33	0.92
		3	−14	0.87	35	0.95
	0.20	0	3	0.94	6	0.94
		1	−4	0.93	18	0.95
		2	−14	0.88	25	0.94
		3	−18	0.83	26	0.94
50	0.10	0	1	0.94	2	0.96
		1	−2	0.94	19	0.94
		2	−6	0.92	40	0.92
		3	−12	0.87	34	0.51
	0.15	0	2	0.93	3	0.95
		1	−6	0.91	16	0.96
		2	−11	0.28	22	0.94
		3	−17	0.79	29	0.91
	0.20	0	3	0.94	1	0.96
		1	−8	0.89	15	0.94
		2	−15	0.82	22	0.92
		3	−24	0.70	22	0.94
100	0.10	0	1	0.93	2	0.95
		1	−4	0.92	18	0.91
		2	−7	0.88	28	0.90
		3	−13	0.78	34	0.86
	0.15	0	2	0.94	3	0.95
		1	−4	0.93	20	0.93
		2	−9	0.86	29	0.89
		3	−14	0.78	32	0.87
	0.20	0	2	0.94	2	0.95
		1	−10	0.86	12	0.95
		2	−19	0.65	16	0.92
		3	−25	0.44	18	0.92

*j is the number of level-2 units; ICC, intra-class correlation; items refers to the number of ignored non-isomorphic items; Rel bias, relative bias expressed as a percentage; Cov, coverage expressed as a proportion*.

## Discussion

Psychometric isomorphism is an important topic in the social sciences, but until recently it has been viewed as a consideration of secondary importance to applied researchers who have emphasized the importance of homology. Recently, research in multilevel modeling has focused on methods for testing different forms of isomorphism in the context of MSEMs (Jak et al., 2013, 2014b; Ryu, 2015, 2014; Kim et al., 2015) as well as the aspects of the research design that produce accurate parameter estimates under different estimation approaches, for example, the number of level-2 clusters required for accurate estimation of parameters using maximum likelihood and Bayesian estimators (Hox et al., 2012, 2014). Until now, however, the connection between psychometric isomorphism and homology in the context of MSEMs has not been thoroughly explored. In this article, we used a Monte Carlo design to explore the impact of ignoring isomorphism on conclusions about homology. We note that our results apply to the condition of higher between level loadings.

### Main findings

The results of this study reveal that the connection is an intimate one. In particular, the direction of the estimation error for the within and between level structural coefficients depends on the degree of the bias in within and between factor loadings and within and between latent variances. Even minor levels of item non-isomorphism cannot be ignored without jeopardizing the accuracy of structural parameter estimates across levels of analysis in MSEM studies. When the item non-isomorphism exists and is ignored on the exogenous measurement model, the within structural coefficient is overestimated and the between structural coefficient is underestimated. When the non-isomorphism exists and is ignored on the endogenous measurement model the within structural coefficient is underestimated and the between structural coefficient is overestimated. In other words, if you fit a model with equality constraints on the non-isomorphic items with categorical data your structural parameter estimates will be biased. In addition, under Bayesian estimation the *ppp*-value will tell you that your model fits. Where detecting non-isomorphism is important and assuming the models will converge it may be advisable to try maximum likelihood approaches (e.g. Jak and Oort, 2015).

Another important finding was despite the high convergence and admissibility rates with Bayesian estimation. The current study showed that even Bayesian estimation has limits with regard to estimation accuracy with very low level-2 sample sizes and low ICCs. This finding affirms the results of Hox et al. (2014) and Hox et al. (2012) who also observed limits on the estimation accuracy of Bayesian methods, even though Bayesian estimation outperformed Maximum Likelihood in their studies in this regard. In this study the smallest ICC condition of 0.05 led to unacceptable relative bias in the between regression parameters even in the correctly specified conditions of the simulation. This estimation error was mitigated but not eliminated by increasing the level-2 sample size to 100 units. It is generally accepted that small ICCs can still warrant Level-2 modeling if the Level-2 factor is of theoretical interest. However, it is advisable that researchers investigating isomorphism and homology with small ICCs get very large sample sizes, i.e., in excess of 100 units. In many cases, this is a difficult task. For instance, when countries are studied the average level-2 sample size is often much lower. In cases such as this, it is recommended that researchers consider adopting a weakly informative or informative prior and examine the sensitivity of the modeling results to the choice of prior by following techniques described, for example, by Depaoli and van de Schoot (2015).

### Limitations and future directions

As one of our reviewers pointed out, if larger factor loadings are at the within level, factor loadings at the within level could be underestimated and factor loadings at the between-level over-estimated. Consequently, to compensate for the lower (higher) loadings, the factor variance at the within level would be overestimated and the factor variance at the between level would be underestimated. This could then have consequences for the structural coefficient in the opposite directions from the current study. This thought experiment highlights that the current conclusions are specific to the situation with larger factor loadings at the between level and non-isomorphism in one of the measurement models.

In terms of methodological limitations, the current study shares certain similar characteristics to the Bayesian Monte Carlo study reported by Hox et al. (2012) in that we were also unable to inspect trace plots for parameter convergence for all models due to many thousands of models that were estimated, but we emphasize inspection of convergence is critical in applied applications. We also undertook extensive further investigations of the extreme cells in our study. We adopted not to use informative priors, preferring not to risk the prior overwhelm the data in our smaller sample conditions. Finally, we also only looked at the case where loadings are higher on the between level.

## Future research directions

The average observed *ppp*-value for just about all cells of the Monte Carlo design met the criterion specified for good fit, including the most misspecified models with high ICCs and largest level-2 sample size. Moreover, the *ppp*-value did not deteriorate with increased ignored non-isomorphism and in some cases improved. It seems important to examine the conditions under which the *ppp*-value, a key model fit criterion in Bayesian estimation of structural equation models, can and cannot be relied on when examining isomorphism in MSEMs with ordinal indicators. For now, based on these results and assuming the models run successfully, more powerful approaches to test isomorphism available in a frequentist framework may be a viable option.

## Author contributions

The author confirms being the sole contributor of this work and approved it for publication.

### Conflict of interest statement

The author declares that the research was conducted in the absence of any commercial or financial relationships that could be construed as a potential conflict of interest.
